# A Case of Herpes Zoster Presented with Lower Limb Paresis

**DOI:** 10.7759/cureus.2923

**Published:** 2018-07-05

**Authors:** Young Rak Choi, Chi Hoon Oh, Wonchul Choi

**Affiliations:** 1 Department of Orthopedic Surgery, CHA University, Cha Bundang Medical Center, Sungnam, KOR; 2 Department of Orthopaedics, CHA University, Cha Bundang Medical Center, Sungnam, KOR

**Keywords:** herpes zoster, varicella zoster virus, lower limb paresis

## Abstract

Herpes zoster is a common viral disorder that typically shows characteristic painful skin lesion. Motor neuropathy rarely complicates herpes zoster infection, and it may be overlooked without suspicion. Here, we report a case of a herpes zoster patient who presented with sciatica and paresis, but without the typical skin lesion. The patient was initially misdiagnosed as having other disorders including trauma or spine lesion. Electrodiagnostic study and magnetic resonance imaging (MRI) helped to make an accurate diagnosis and localize the motor nerve involvement of herpes zoster.

## Introduction

Herpes zoster is a viral disease that results from the reactivation of varicella zoster virus (VZV). It is a relatively common disorder that the cumulative lifetime incidence of herpes zoster reaches 10%-20% of the population [1]. In general, it is characterized by skin eruptions and pain in specific dermatomes, and it is frequently complicated by residual pain called post-herpetic neuralgia [2].

On the other hand, motor nerve involvement of VZV is infrequently complicated, so the reported incidence is less than 5% [3]. Moreover, the symptom may mimic sciatica or weakness related to a spine root compressive disorder, so the diagnosis can be difficult without suspicion. The diagnosis can be even more challenging if the motor paresis presents without the typical skin lesion related to herpes zoster.  

Here, we report a case of a herpes zoster patient who presented with sciatica and paresis but without the typical skin lesion, which was misdiagnosed initially.

## Case presentation

A 48-year-old female patient visited the emergency room (ER) due to left buttock pain after she slipped down on her way to the bathroom. The accident occurred just before she visited the ER, while the left buttock pain started two weeks ago. The patient denied for any remarkable medical history except for being a hepatitis B virus carrier. According to the ER records, she had a generalized fever up to 37.8°C and had tenderness, swelling on her left buttock and proximal area of the posterior thigh. The blood laboratory test showed elevated C-reactive protein (CRP) level (4.08 mg/dl) without leucocytosis (WBC: 6930/μl). The liver enzyme values were mildly elevated such that serum glutamic oxaloacetic transaminase (GOT) and glutamic pyruvic transaminase (GPT) were 68 and 45, respectively. The plain radiography showed no evidence of fracture around her hip joint. The emergency medicine physician had a clinical impression of early stage cellulitis or contusion of hip and she was discharged from ER after prescribing empirical antibiotics and nonsteroidal anti-inflammatory medications.

**Figure 1:**
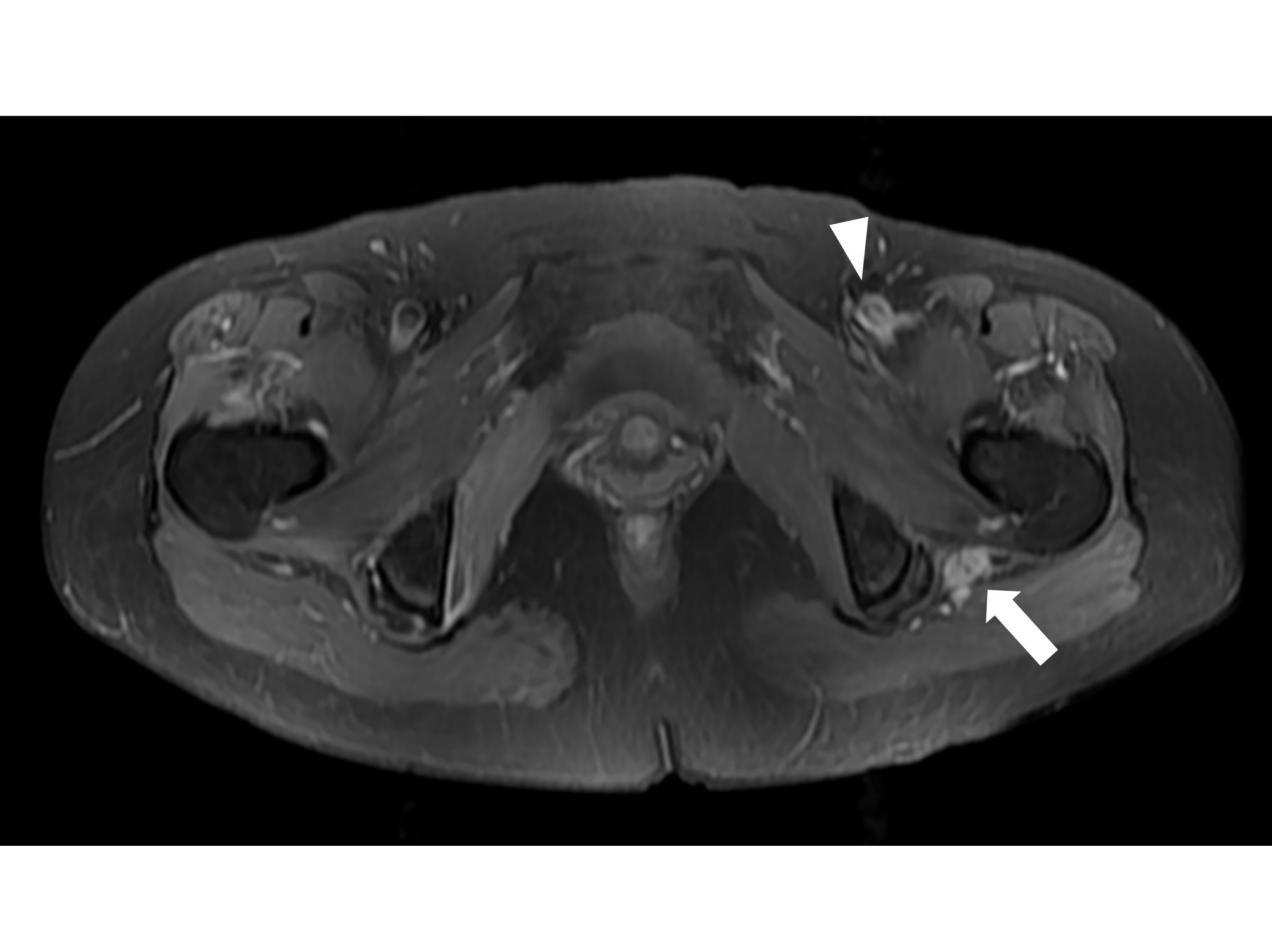
Initial magnetic resonance image (MRI) finding. In T2-weighted axial image after contrast enhancement,  asymmetrically diffuse swelling with increased signal intensity and contrast enhancement of the left sciatic nerve (arrow) was observed. Also mild swelling with T2-hyperintensity of the left femoral nerve (arrowhead) was found in the same plane.

A week later, she visited the orthopedic outpatient department with aggravated pain and weakness on her left lower extremity. The vital signs were unremarkable. On physical examination, she had a left foot drop and was unable to dorsiflex or plantar-flex her left ankle. Extension of the great toe was also impossible. Hypoesthesia was detected on L4, 5, S1 dermatomes. Deep tendon reflexes on patellar and Achilles tendon were normal and symmetrical. A patchy erythematous rash with sharp pain existed on the left buttock and posterior thigh without any sign of vesicle formation. The blood laboratory test showed mildly elevated CRP (2.03 mg/dl) and normal procalcitonin (0.05 ng/ml) levels. Due to the aggravated sciatica and left lower limb weakness, the lumbosacral magnetic resonance imaging (MRI) was urgently performed under suspicion of a spinal lesion. The MRI revealed mild bulging disc with bilateral facet arthritis at L4-5 level; however, it was not thought to be the direct cause of the symptoms. The result of electromyography and nerve conduction velocity (EMG-NCV) tests showed severe left sciatic neuropathy that no motor nerve responses from the left peroneal nerve and reduced compound muscle action potential on tibial nerve were found. Pelvis MRI with contrast enhancement revealed asymmetrical diffuse swelling of the left sciatic and femoral nerve, which suggested neuritis (Figure 1).

**Figure 2:**
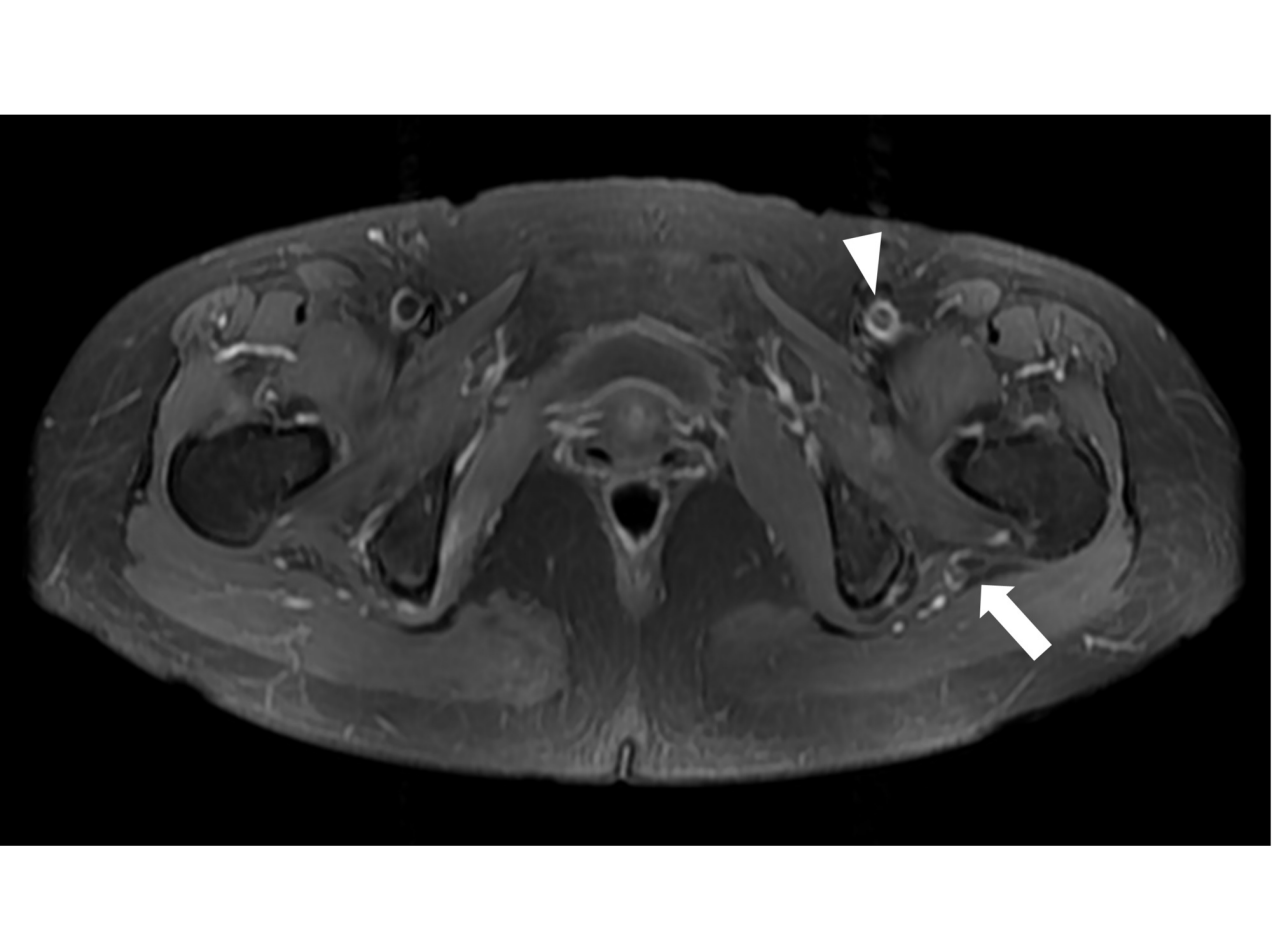
Follow-up MRI finding. Swelling and contrast enhancements of sciatic (arrow) and femoral (arrowhead) nerves were decreased after treatment.

A more detailed investigation on her past medical history revealed that she had been treated for herpes zoster infection of the left buttock three years ago. A thorough systematic review was done again; however, she had no comorbidity or medication history that can cause immunosuppression. The putative diagnosis of herpes zoster was confirmed by detecting VZV DNA from her blood sample using the polymerase chain reaction test. The patient was treated with famciclovir 500 mg every eight hours and 20 mg/day of prednisolone for two weeks and changed to oral pregabalin 75 mg every 12 hours thereafter. After 12 weeks of an inpatient rehabilitation admission, she had a partial neurological recovery and could walk with a foot drop splint. The manual muscle test showed that muscular strength of her left ankle dorsi-flexor, plantar-flexor and great toe extensor improved to grades II, III, and III, respectively. The hypoesthesia of S1 dermatome was completely recovered, while L4, L5 dermatomes showed 50% improvement. Also, the skin rash and pain around it was completely alleviated. Follow-up pelvis MRI taken at 10 weeks after the treatment showed markedly diminished swelling of the left sciatic and femoral nerve (Figure 2). 

## Discussion

Herpes zoster is the common manifestation of the VZV reactivation. It is usually presented as a self-limiting vesicular rash, often accompanied by post-herpetic neuralgia [3]. Though relatively rare, the virus can affect the corresponding myotome and cause motor weakness or paresis after VZV reactivation [4-6]. If the virus affects the lower motor nerve, the symptom may mimic sciatica or paresis caused by the lumbar spine lesion [7-10]. Moreover, the weakness or paresis can infrequently occur in the absence of the typical skin lesion associated with herpes zoster and make it extremely difficult to diagnose [11-13]. The patient of this case report had sciatica before the typical herpes zoster skin lesion developed. Also, it is highly suspected that she had preceded lower limb paresis which caused the slip-down accident. Unfortunately, neurological examination was neither performed nor documented during her ER visit, and an accurate diagnosis may have been delayed.

The localization of motor abnormality can be also challenging because the involved myotomes do not always correspond to the dermatomes affected by a zoster skin rash [14]. The EMG-NCV tests have been generally used to evaluate the extent of herpes zoster-associated neuropathy [4, 12-13, 15]. However, the accuracy of those studies may vary depending on the physician’s skill and often difficult to test immediately. On the other hand, MRI could be useful to localize the causative lesion [6, 14]. Our case showed abnormal nerve findings on MRI, and it was unique that both sciatic and femoral nerves were affected. Interestingly, Jones et al. [16] have reported the largest series of zoster-associated limb paresis and only one of four zoster-associated lumbosacral plexopathy or radiculopathy showed MRI abnormalities while MRIs of all four mononeuropathy cases had abnormal findings on nerves. Regarding this, MRI may be more helpful to diagnose the distal neuropathy related to VZV infection.

Herpes zoster usually occurs in the immunocompromised patient. Although the patient of our case had a history of previous VZV infection, her past medical history was unremarkable. We are not sure whether being a hepatitis B virus carrier resulted in the VZV reactivation.

Favorable prognosis of herpes zoster-associated motor paresis has been generally reported [7, 9-10], while prolonged weakness may happen [6, 13, 15]. The prognostic factor is still unclear yet, but the risk of the persisting symptom may get higher if treatment is delayed [17]. Also, it was postulated that poorer recovery is expected when motor neuropathy precedes the rash, as in our case, than when motor neuropathy follows the rash [12]. The patient in our case had only a partial recovery of paresis until three months after treatment, so longer-term follow up with continuous rehabilitation would be necessary.

## Conclusions

Herpes zoster-associated lower limb paresis may be an uncommon complication of a common disorder. The diagnosis can be challenging as sciatica or weakness may occur before the typical herpes zoster skin lesions develop. Also, the relation between the skin lesion and the motor symptom may be overlooked. Physicians should be aware that the zoster-associated motor paresis can mimic a compressive root lesion in its presentation. We believe it should be considered as a differential diagnosis of lumbosacral spine lesions, especially in debilitated patients, even if the typical skin lesion is absent. Thorough investigation of the past medical history, accurate neurological tests, and exploration for the skin lesion would be essential. A combined use of EMG-NCV and MRI may help to diagnose and localize the affected site.
